# A gene expression signature‐based nomogram model in prediction of breast cancer bone metastases

**DOI:** 10.1002/cam4.1932

**Published:** 2018-12-21

**Authors:** Chenglong Zhao, Yan Lou, Yao Wang, Dongsheng Wang, Liang Tang, Xin Gao, Kun Zhang, Wei Xu, Tielong Liu, Jianru Xiao

**Affiliations:** ^1^ Spine Tumor Center, Department of Orthopedic Oncology, Changzheng Hospital Second Military Medical University Shanghai China

**Keywords:** bone metastases, breast cancer, GSEA, nomogram, prognosis

## Abstract

Breast cancer is prone to form bone metastases and subsequent skeletal‐related events (SREs) dramatically decrease patients’ quality of life and survival. Prediction and early management of bone lesions are valuable; however, proper prognostic models are inadequate. In the current study, we reviewed a total of 572 breast cancer patients in three microarray data sets including 191 bone metastases and 381 metastases‐free. Gene set enrichment analysis (GSEA) indicated less aggressive and low‐grade features of patients with bone metastases compared with metastases‐free ones, while luminal subtypes are more prone to form bone metastases. Five bone metastases‐related genes (KRT23, REEP1, SPIB, ALDH3B2, and GLDC) were identified and subjected to construct a gene expression signature‐based nomogram (GESBN) model. The model performed well in both training and testing sets for evaluation of breast cancer bone metastases (BCBM). Clinically, the model may help in prediction of early bone metastases, prevention and management of SREs, and even help to prolong survivals for patients with BCBM. The five‐gene GESBN model showed some implications as molecular diagnostic markers and therapeutic targets. Furthermore, our study also provided a way for analysis of tumor organ‐specific metastases. To the best of our knowledge, this is the first published model focused on tumor organ‐specific metastases.

## 1 INTRODUCTION

Breast cancer (BC) is the most common malignancy among women patients worldwide.^1^, ^2^ With the advances achieved in early diagnosis and effective management of primary tumors, the overall survival of BC has increased in recent decades whereby metastases have become the leading cause of death.^3^ Bone is a common site of metastases, and BC is one of the most common cancers to form metastatic bone lesions.^4^ Most of bone metastases of BC patients were osteolytic, which could cause skeletal‐related events (SREs) including bone destruction, pathological fractures, hypercalcemia, and spinal cord or nerve root compression.^5^, ^6^ Once bone metastases were diagnosed, the overall survival of BC patients decreased dramatically and median life expectancy dropped to 2‐3 years.^4^, ^7^ Current treatment options for BC bone metastases (BCBM) are seldom curative and are instead mostly palliative.^8^ Surgical resection and stabilization are effective for solitary lesions; however, patients with multiple metastases or bad general conditions are not suitable for surgical interventions.^9^ Therefore, prediction and early detection of BCBM are valuable.

Breast cancer is a molecularly heterogeneous disease which similar tumors form different clinical outcomes and metastases patterns.^10^ Smid et al^11^ reported that different subtypes of BC show different preferential sites of relapse; bone relapse were most abundant in the luminal subtypes but were found less than expected in the basal subtype. Effective therapeutic intervention relies on a better mechanistic understanding of metastasis organotropism. Kang et al^12^ revealed a complex but sophisticated multi‐genic program mediating breast cancer metastasis to bone, while Zhuang et al^13^ reported that BCs with high expression of DKK1 are prone to form bone metastases while low expression of DKK1 correlated with lung metastases. All these findings indicated that the gene expression patterns of primary tumor could predict patients’ prognosis or bone metastases to a certain extent.

The high‐throughput microarrays have emerged as a promising and efficient tool for studies of the complex pathogenesis of human diseases including cancer. Meanwhile, repurposing and collected analyzing of microarray data for reveal of tumor formation mechanisms and survival prediction are well established. Guo et al^14^ collected four publically available gene expression datasets including 319 colorectal cancer and 103 normal tissues, and successfully identified critically functional genes and pathways in tumor progression. Pan et al^15^ constructed a five‐gene‐based prognostic model for colorectal cancer based on four gene expression datasets while Meng et al^16^ constructed a four‐long non‐coding RNA signature in predicting breast cancer survival based on 887 patients from three microarray datasets.

In the current study, we reviewed a total of 572 BC patients in three microarray datasets including 191 bone metastases and 381 metastases‐free. One hundred and one differentially expressed genes (DEGs) were identified, 21 of which are highly correlated with bone metastases. With the random survival forests algorithm and multivariate Cox regression analysis, we constructed a five‐gene (KRT23, REEP1, SPIB, ALDH3B2, and GLDC) expression signature‐based nomogram (GESBN) model. The stratification based on the prognostic value of the GESBN model was further validated in the two independent testing sets. Altogether, these results indicated the potential value of our model for clinical evaluation and prediction of bone metastases. To the best of our knowledge, this is the first organ‐specific prediction model for tumor metastases.

## 2 MATERIALS AND METHODS

### 2.1 GEO breast cancer gene expression data

Breast cancer microarray datasets were obtained from the Gene Expression Omnibus (GEO; http://www.ncbi.nlm.nih.gov/geo/). The datasets with more than 50 primary breast tumors characterized with bone metastases information were selected for further analyses. Dataset with GEO accession number GSE12276 (platform GPL570) was used as a training set to identify DEGs between bone metastases and bone metastases‐free patients.^17^ While GSE2034 and GSE2603 (platform GPL96) were used as testing sets for independent validation.^18^, ^19^

### 2.2 Gene set enrichment analysis (GSEA)

Gene set enrichment analysis was performed by the javaGSEA Desktop Application (http://software.broadinstitute.org/gsea/downloads.jsp) using MSigDB C2: curated gene sets (4762 gene sets available). Gene sets with a false discovery rate (FDR) value <0.05 after performing 1000 permutations were considered to be significantly enriched.^20^ Enrichment Map was used for visualization of the GSEA results.

### 2.3 Microarray data processing and nomogram generation

All downloaded microarray data were processed by R software version 3.5.0 using packages from Bioconductor (https://cran.r-project.org/src/base/R-3/R-3.5.0.tar.gz). Raw data were first normalized using the robust multi‐array average (RMA) method.^21^ Annotations for probe arrays were downloaded from the GEO database. The genes shared by both the training and validation datasets were selected for further analyses. DEGs between bone metastases and metastases‐free patients in the training set were identified and confirmed for bone metastases‐free survival (BMFS) analysis. The random survival forests model was then used to identify candidates by their relative importance. By multivariate Cox regression analysis, five genes were identified as independent prognostic factors for BCBM and a gene expression signature‐based nomogram (GESBN) model was constructed for prediction of BCBM.

### 2.4 Statistical analysis

The association between the filtered DEGs and BMFS was assessed by univariate Cox regression analysis in the training set. Twenty‐one genes with *P* value <0.05 were considered as significant ones. These genes were further subjected to the random survival forests model. With a cut‐off of relative importance >0.5, eight genes were selected and incorporated into the multivariate Cox regression model, five of which were finally identified as independent factors for BCBM. Receiver operating characteristic (ROC) curves were drawn and area under curves (AUC) were calculated for each gene and their combination.

We then constructed a GESBN model for prediction of BCBM based on the five genes. The performance of the nomogram was measured by concordance index (C‐index). All patients in the training and testing sets were assigned with a GESBN score and further divided into high‐risk and low‐risk groups using the median score as the cut‐off. Patients having higher scores are expected to have higher risks of bone metastases. Differences of BMFS between groups were compared using the Kaplan‐Meier method. The prediction accuracy of nomogram was further assessed by calibration plots for the probability of survival at 3‐ or 5‐year. All statistical analyses were implemented in R software with significance level at 0.05.

## 3 RESULTS

### 3.1 Characteristics of breast cancer bone metastases

A total of 572 breast cancer patients, consisting of 204 in the GSE12276 training set, 286 in the GSE2034 testing set and 82 in the GSE2603 testing set, were included in the current studies (Detailed in Table S1). One hundred and eleven patients in the training set and 80 in the testing set suffered bone metastases. The incidence of bone metastases ranges from 13.4% to 54.4% across datasets.

**Figure 1:**
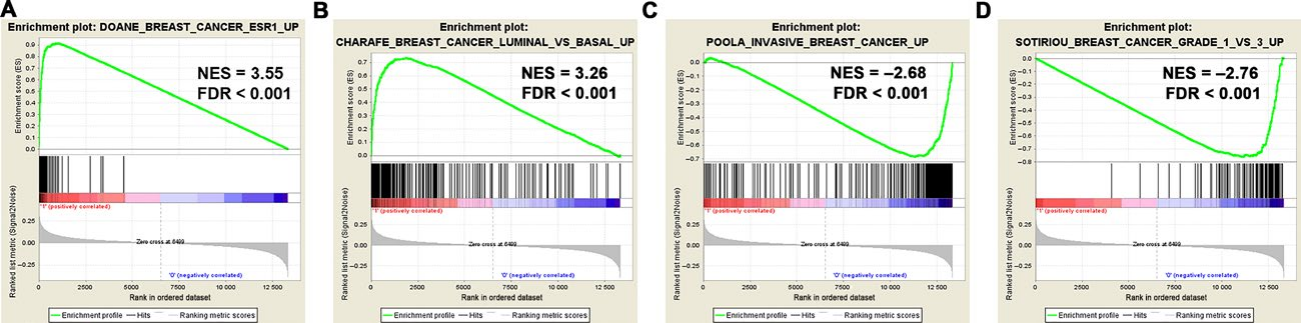
Gene set enrichment analysis (GSEA) delineates the clinical characteristics of BCBM. Patients with BCBM are characterized as ESR activated (A) and the Luminal subtype signature (B). Meanwhile, they showed features of less aggressiveness (C) and low tumor grade (D)

To further reveal characteristics of BCBM, we performed the GSEA between bone metastases and metastases‐free patients in the training set, and observed an Estrogen receptor (ESR) activated and the Luminal subtype signatures were closely associated with BCBM (Figure 1A,B), in accordance with published literatures that the luminal subtypes are more prone to form bone metastases.^10^, ^11^ Meanwhile, these results indicated that bone metastases more frequently occurred in less aggressive and low‐grade patients (Figure 1C,D and Figure S1).

### 3.2 Construction of the gene expression signature‐based nomogram model

**Figure 2:**
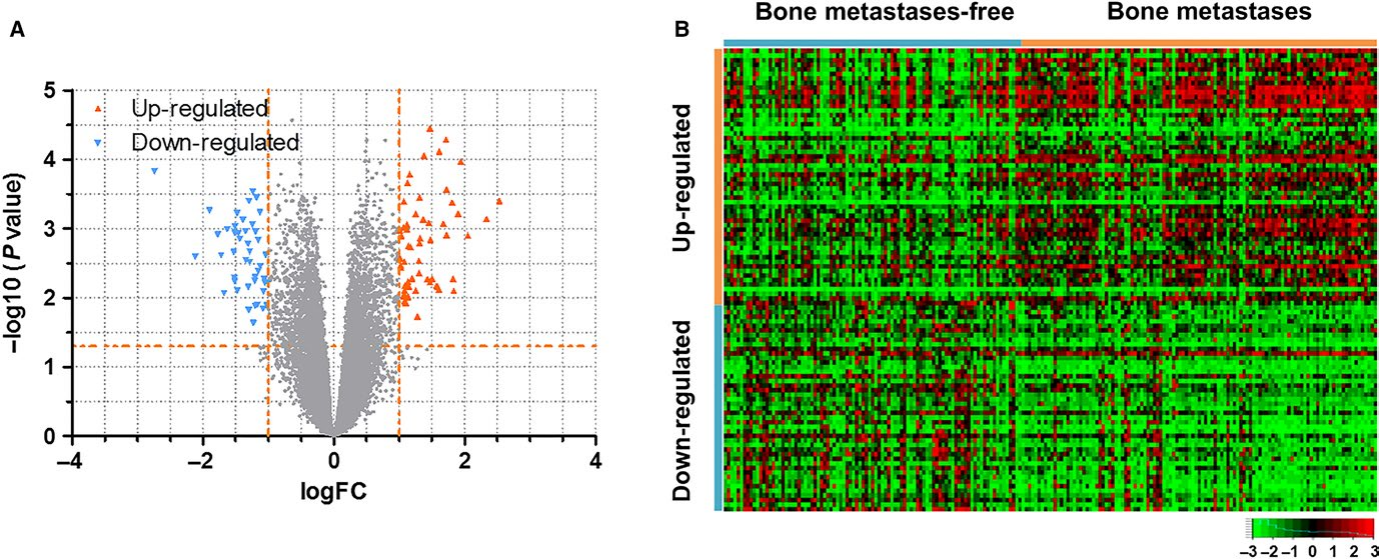
DEGs between bone metastases and metastases‐free patients. A, Volcano plots showed 56 up‐regulated (red plots) and 45 down‐regulated (blue plots) genes. B, Heat maps of the 101 DEGs

With the threshold of *P* < 0.05 and [logFC] >1, 101 DEGs were identified in the training set including 56 up‐regulated and 45 down‐regulated genes (detailed in Table S2). Volcano plots and heat maps of the 101 DEGs are shown in Figure 2. DEGs were further subjected to the univariate Cox proportional hazard regression model and 21 survival‐related DEGs were identified (detailed in Table S3).

**Figure 3:**
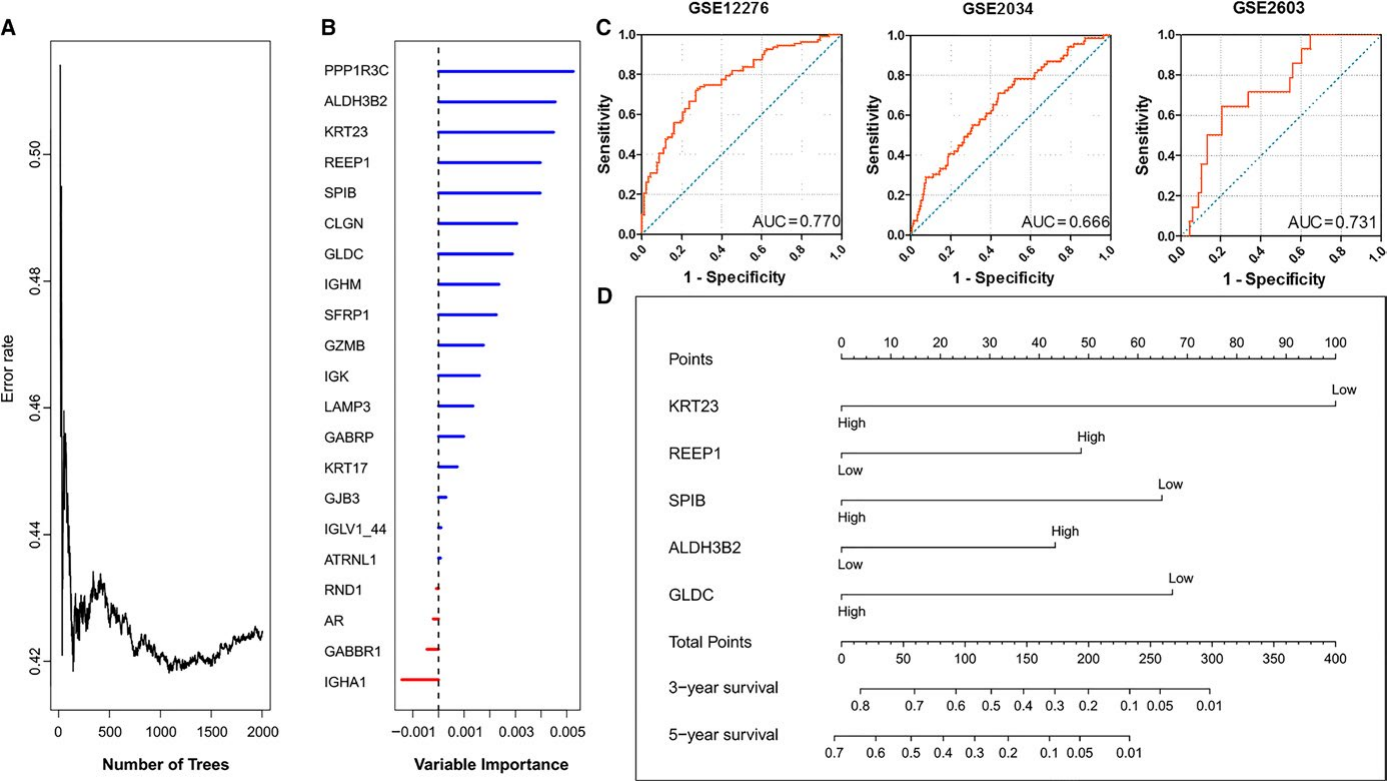
Construction of the Gene Expression Signature‐Based Nomogram (GESBN) Model. A, Error rate of random survival forests algorithm. B, Variable importance of the 21 survival‐related DEGs. C, Receiver operating characteristic (ROC) analysis of the five‐gene signature in the training (GSE12276) set and testing (GSE2034 and GSE2603) sets. D, The five‐gene‐based GESBN Model

To select the most weighted genes, we used the random survival forest model and ranked these 21 survival‐related DEGs by their relative importance (Figure 3A,B and Table S4). Eight genes, including PPP1R3C, KRT23, ALDH3B2, REEP1, SPIB, CLGN, GLDC, and IGHM, were selected as the most important candidates (relative importance >0.5) and further subjected to the multivariate Cox regression model.

Subsequently, KRT23, REEP1, SPIB, ALDH3B2, and GLDC were identified as independent prognostic factors for BCBM by multivariate Cox regression analysis (Detailed in Table S5). Although PPP1R3C had a higher importance value than others, it was still not included in the multivariate Cox regression model. ROC analysis of the five genes further demonstrated their effectiveness in predicting BCBM (Figure S2). The BCBM prediction model under the five‐gene signatures showed higher performance in both the training (GSE12276) set and testing (GSE2034 and GSE2603) sets (Figure 3C) as compared with the model under each of the five genes. Therefore, a five‐gene expression signature‐based nomogram model (GESBN) was constructed for BCBM prediction (Figure 3D).

### 3.3 Evaluation and validation of the GESBN model

**Figure 4:**
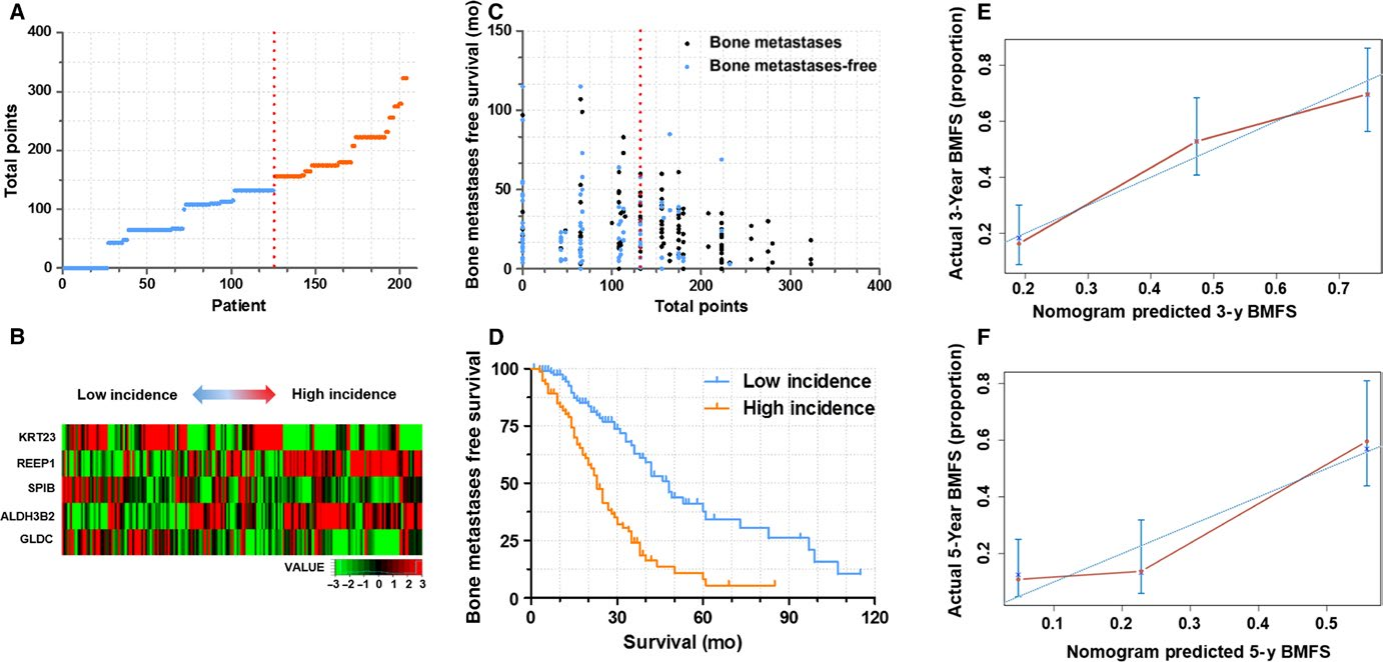
GESBN Model analysis of Patients in the training set (GSE12276). A, GESBN score distribution. Red line indicated the median score. B, Heat map of the five‐gene signature. Patients were arranged by GESBN scores from low incidence to high incidence. C, Bone metastases status and survival time. Red line indicated the median score. D, Kaplan‐Meier survival curves between high incidence and low incidence patients. E and F, The calibration curves for predicting patient BMFS at 3‐ and 5‐year (Internal verification)

We first calculated the GESBN score for each patient in the training set. Patients were ranked according to their risk scores and divided into two groups as low‐risk and high‐risk of bone metastases based on the five‐gene signature (As shown in Figure 4A,B). Patients in the high‐risk group had significantly shorter bone metastases‐free survival than those in the low‐risk group (log‐rank test, *P* < 0.0001, Figure 4C,D). The C‐index for BMFS prediction was 0.677. The calibration plot for the probability of survival at 3‐ or 5‐year showed a good agreement between the prediction by nomogram and actual observation (Figure 4E,F).

**Figure 5:**
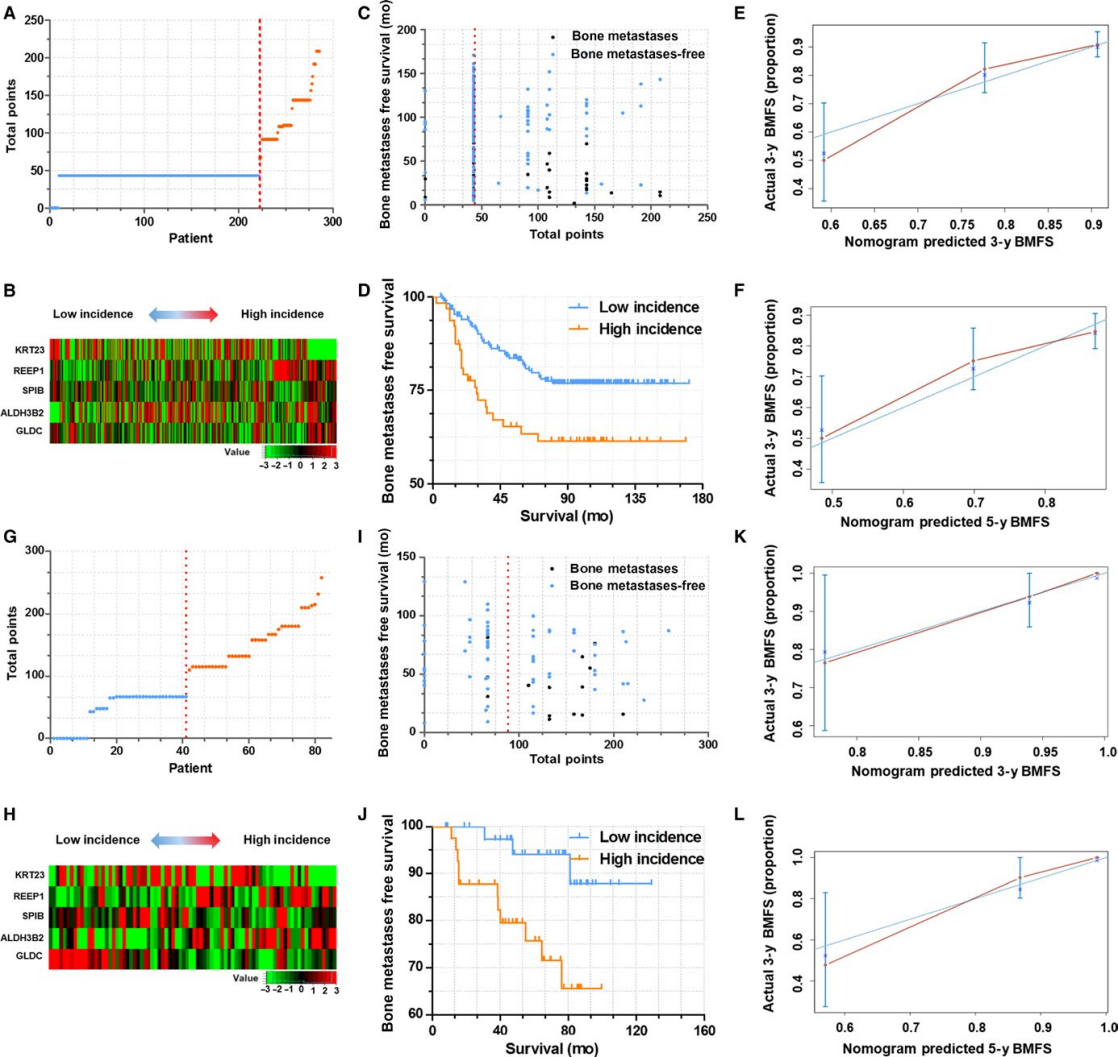
GESBN Model analysis of Patients in the testing set. A‐F, Validation of GESBN model in dataset GSE2034. G‐L, Validation of GESBN model in dataset GSE2603

To further confirm the predictive value and clinical significance, we calculated the GESBN score for patients in the two testing sets of GSE2034 and GSE2603. Significant differences of BMFS between high‐risk and low‐risk groups were observed in both of the two testing sets（Figure 5A‐D,G‐J）. Meanwhile, the C‐indices of the nomogram for predicting BMFS were 0.689 and 0.695 for GSE2034 and GSE2603 respectively. In accordance with the training set, calibration curves also showed good agreement between prediction and observation in the probability of 3‐ and 5‐year survival for both sets (Figure 5E‐F,K‐L).

### 3.4 Functional analysis of the two prognostic subtypes in breast cancer

**Figure 6:**
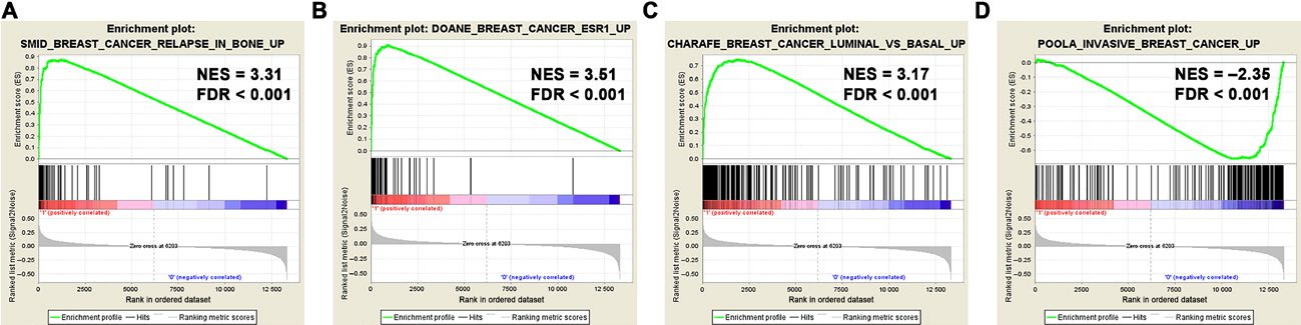
GSEA of patients between high and low GESBN scores. Patients with high GESBN scores showed similar GSEA signatures in accordance with patients who suffered bone metastases (listed Figure 1 and Figure S1)

Finally, we performed the GSEA analysis between patients of high‐risk and low‐risk groups in the training set (Figure 6 and Figure S3). Patients with high GESBN scores are featured with a signature of breast cancer relapse in bone. Besides, similar GSEA signatures, such as ESR activation, Luminal subtypes and invasive breast cancer signatures are well characterized and are in accordance with patients who suffered bone metastases (listed in Figure 1 and Figure S1). These findings further confirmed the validity and reliability of our GESBN model for clinical evaluation and prediction of bone metastases.

## 4 DISCUSSION

BC is one of the cancers that most commonly metastasize to bone. It is reported that ~50% of patients with advanced disease will develop clinically detectable osteolytic bone metastases while the incidence increases to over 75% by autopsy.^22^ Luminal subtypes of cancer, which are considered of relatively good prognosis, are more prone to form bone metastases.^10^, ^11^ Meanwhile, unlike other sites of metastases, such as lung, liver, and brain, bone metastases were considered as less lethal.^23^ In the current study, we compared the gene set enrichment features of patients with or without bone metastases. BCBM owned the Luminal subtype signatures and showed less aggressive and low‐grade features. All these findings indicated that although suffering bone metastases, patients’ tumor is still of low malignancy and the prognosis could be better if the bone lesions are well controlled.

Bone lesions seldom lead to death directly. However, the survival of patients with BCBM decreased dramatically.^24^ The largest barrier to a good outcome in bone metastases is the lack of appropriate treatment strategies in management with tumor‐induced SREs.^25^ Bone metastases often present as challenges since the therapies, which effectively developed for primary tumors, are unsatisfying when subjected to patients with bone metastases.^3^ Surgical interventions are proved to show some value for survival improvement, while patients with multiple lesions are not always suitable for surgery.^24^ Therefore, identifying risk factors and developing predictive models of bone metastases for primary tumor patients are valuable. By such risk factors and/or models, early detection and interventions become realistic.

Clinical studies concerning on risk factors of BC bone metastases were limited.^26^ Our previous study identified that lymph node metastases and ESR status were independent risk factors in predicting BC spine metastases.^27^ Besides, Chen et al^28^ indicated that axillary lymph node metastases and the concentrations of CA125, CA153, ALP, and hemoglobin were independent risk factors for BCBM. Several studies have reported gene signature‐based prognostic prediction models for BC patients by repurposing and analysis of microarray data.^29^, ^30^ These models were constructed based on primary tumor gene expression characteristics and concerned about patients’ overall survival or local recurrence. However, models in the prediction of bone metastasis are missing. By reviewing a total of 572 BC patients, we constructed GESBN model based on five genes for prediction of BCBM. To the best of our knowledge, this is the first published model for evaluation and prediction of tumor organ‐specific metastases based on gene expression signatures.

KRT23 is a type I acute‐phase responsive gene and was identified to encode a member of the keratin family, which serves as the structural proteins in epithelial cells.^31^ Previous studies showed that KRT23 knockdown decreases proliferation and affects the DNA damage response of colon cancer cells, and KRT23 represents a specific, stress‐inducible ductular reaction marker.^32^, ^33^ REEP1 protein, which is preferentially expressed in neuronal and neuronal‐like exocytotic tissues like brain, spinal cord, and testes and localized to endoplasmic reticulum (ER) and plasma membranes, is a member of a family of ER shaping proteins.^34^, ^35^ It has been found that REEP1 could facilitate mitochondrial‐ER interactions, which may result in intracellular Ca^2+^ overload and axonal damage,^36^ and REEP1 variants were recognized as causes of Neurological Disease like hereditary spastic paraplegia (HSP) and distal hereditary motor neuropathy (dHMN).^37^ The third gene SPIB, encoding an ETS‐domain transcription factor, was believed to be associated with cancers especially in lymphomas.^38^, ^39^ Also, Yi‐Jung et al^40^ found the SPIB may be involved in tumorigenesis in live cancer, and Wei et al^41^ reported that SPIB is expressed in invasive cancer cells in human primary lung cancer tissues. ALDH3B2 was widely known as a member of aldehyde dehydrogenases (ALDHs).^42^ It plays a major role in the detoxification of aldehydes generated by alcohol metabolism and lipid peroxidation and has been implicated as causes of some cancers and male infertility.^43^, ^44^ The last gene GLDC, which encodes glycine decarboxylase. It serves as an oncogene that promotes tumorigenesis and cellular transformation.^45^ Several studies have reported that GLDC was overexpressed in many cancer cell lines, including ovarian, cervical, lung, lymphoma, prostate, and phyllodes cancer cell lines.^46^, ^47^ Moreover, Sabina et al^48^ found that GLDC expression is exploited by malignant tumors for adapting their metabolism under hypoxic conditions, thereby being associated with aggressiveness.

The five genes were weighted by the multivariate Cox regression model and further subjected to the nomogram scoring model. Nomograms have been developed and shown to be more accurate than the conventional staging systems for predicting prognosis in some cancers.^49^, ^50^ In the current study, the GESBN model performed well in predicting BCBM, and its prediction was supported by the C‐index (0.677 for training set and 0.689 and 0.695 for testing sets, respectively) and the calibration curve.

There are still some limitations in the current study. First, the training set and testing sets are of different platforms and only the shared genes (22283 out of 54675) were included in the analysis. Therefore, the prognostic genes identified here may not represent all the candidates that are potentially correlated with BCBM. Secondly, the mechanisms of these five genes in regulation of BCBM remain elusive and still require further studies. Finally, although recapitulated in two published testing sets, prospective clinical trials are still needed for validation. Despite these drawbacks, however, the significant and consistent correlation of our GESBN model with BCBM in several independent data sets indicates that it is a potential and powerful tool for clinical evaluation.

## 5 CONCLUSIONS

In summary, we retrospectively identified five bone metastases‐related genes (KRT23, REEP1, SPIB, ALDH3B2, and GLDC) and constructed a gene expression signature‐based nomogram (GESBN) model for breast cancer patients by bioinformatics analysis. The model performed well in both training and testing sets for evaluation of BCBM. Clinically, the model may help in the early prediction of bone metastases, prevention and management of SREs, and even prolong survivals for patients with BCBM. The five‐gene GESBN model showed some implications as molecular diagnostic markers and therapeutic targets. Furthermore, our study also provided a way for analysis of tumor organ‐specific metastases.

## CONFLICT OF INTEREST

No potential conflicts of interest were disclosed.
