# Supplementary figures and images for: A gene expression signature‐based nomogram model in prediction of breast cancer bone metastases
Source: Cancer Med. 2018 Dec 21;8(1):200–8. doi: 10.1002/cam4.1932 (PMC6346244; doi:10.1002/cam4.1932)

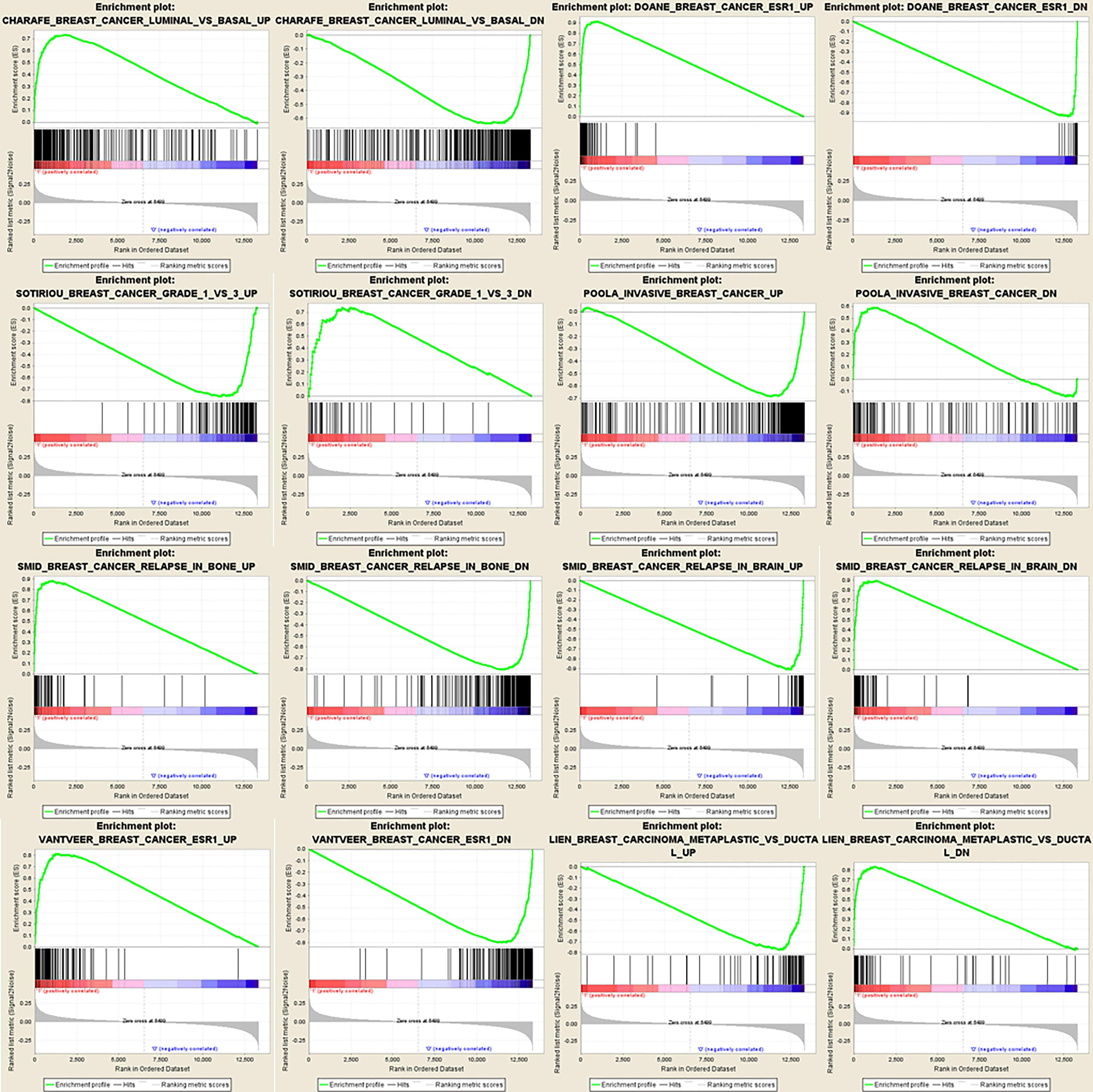

Supplement: Supplementary file 1 [file CAM4-8-200-s001.tif]

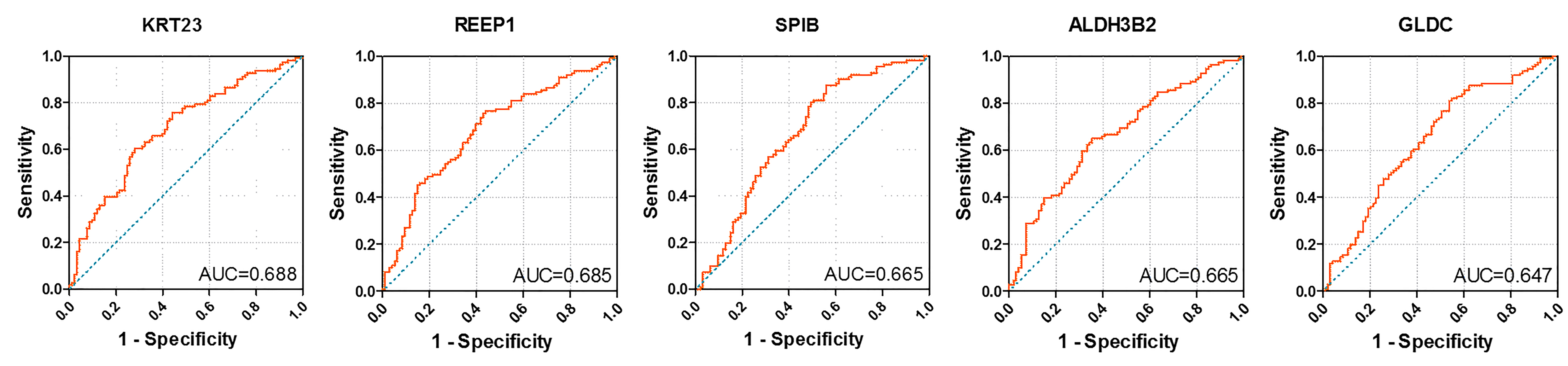

Supplement: Supplementary file 2 [file CAM4-8-200-s002.tif]

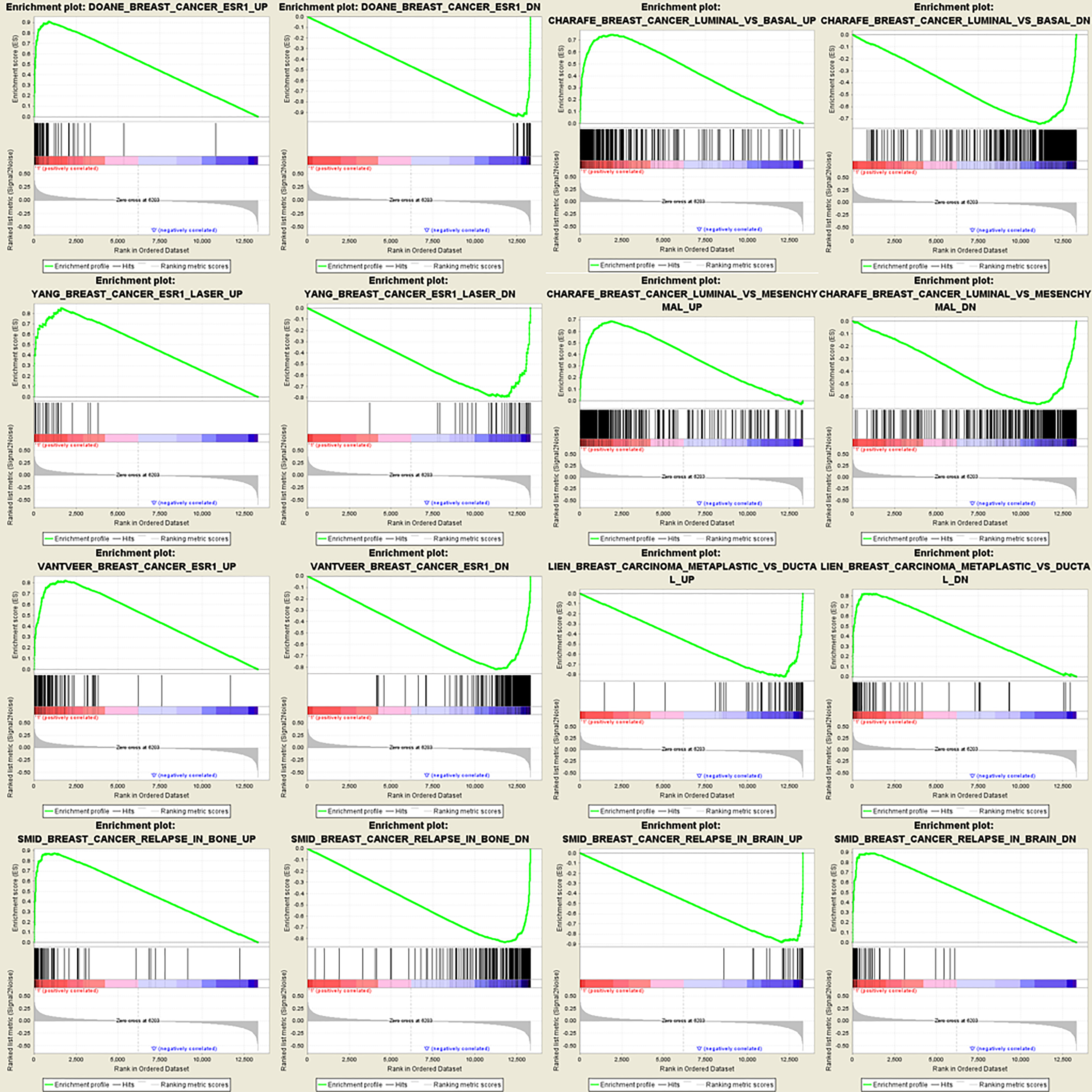

Supplement: Supplementary file 3 [file CAM4-8-200-s003.tif]
