# Supplementary material for: A gene expression signature‐based nomogram model in prediction of breast cancer bone metastases
Source: Cancer Med. 2018 Dec 21;8(1):200–8. doi: 10.1002/cam4.1932 (PMC6346244; doi:10.1002/cam4.1932)
Supplement: Supplementary file 4 [file CAM4-8-200-s004.docx]

Supplemented Table 1.Characteristics of the three datasets.

| **Characteristics** | **GSE12276 (n=204)** | **GSE2034 (n=286)** | **GSE2603 (n=82)** |
| --- | --- | --- | --- |
| **Age, years** | | | |
| Mean (SD) |  | 54 | 56 |
| ⩽55 |  | 165 (58%) | 42 (51%) |
| >55 |  | 121 (42%) | 40 (49%) |
| **Bone Metastasis Event** | | | |
| Yes | 111 (54) | 66 (23%) | 14 (17%) |
| No | 93 (56) | 220 (77%) | 68 (83%) |
| **Lymph Nodes** | | | |
| Positive |  |  | 57 (70%) |
| Negative |  |  | 25 (30%) |
| **ER status**[^*^](https://www.sciencedirect.com/science/article/pii/S0140673605179471?via%3Dihub#tbl1fn1) | | | |
| Positive |  | 209 (73%) | 46 (56%) |
| Negative |  | 77 (27%) | 36 (44%) |
| **PR status**[^*^](https://www.sciencedirect.com/science/article/pii/S0140673605179471?via%3Dihub#tbl1fn1) | | | |
| Positive |  | 165 (58%) | 36 (44%) |
| Negative |  | 111 (39%) | 46 (56%) |
| Unknown |  | 10 (3%) |  |

Supplemented Table 2. DEGs between bone metastases and metastases free patients in GSE12276

| **DEGs** | **Genes names** |
| --- | --- |
| **Upregulated** | AGR2, CYP2B7P, PIP, SLC44A4, ERBB4, DHRS2, TFF1, TSPAN1, ESR1, FOXA1, NAT1, CA12, MLPH, MSMB, TFF3, HMGCS2, CYP4B1, ATRNL1, CHAD, KRT17, PTPRT, SCUBE2, CAPN9, SPDEF, GATA3, LIN7A, HPX, TOX3, ANXA9, ALDH3B2, RET, NAV3, ACOX2, THPO, PSD3, CLGN, C9orf116, SCNN1A, MAPT, AR, REEP1, RND1, STK32B, PP14571, OGN, C4A, MSX2, TBC1D9, OMD, GRP, PPP1R3C, ABAT, ADAM2, CCDC170, SIDT1, GABBR1 |
| **Downregulated** | IDO1, LCN2, GZMB, GLDC, ANXA8, TRIM29, GLV1-44, KLK5, CXCL1, CDH3, KIAA1324, IGHA1, IL12RB2, SPIB, MSLN, SFRP1, MARCO, IGHM, LAMP3, GJB3, MIA, SLC34A2, RYR1, GK, S100B, BBOX1, GF2BP3, FERMT1, RARRES1, CRABP1, GABBR2, KRT5, BCL11A, MMP12, NRTN, SERPINB5, KRT81, KRT16, ROPN1B, FABP7, KLK6, KRT6B, KRT23, VGLL1, GABRP |

Supplemented Table 3. Univariate cox analysis of survival-related DEGs.

| **Gene** | **p-Value** | **HR** | **Gene** | **p-Value** | **HR** |
| --- | --- | --- | --- | --- | --- |
| PPP1R3C | 0.001 | 1.225 | CLGN | 0.018 | 1.135 |
| ALDH3B2 | 0.002 | 1.175 | GZMB | 0.019 | 0.836 |
| IGK | 0.004 | 0.865 | GLDC | 0.019 | 0.761 |
| IGHM | 0.006 | 0.863 | AR | 0.02 | 1.18 |
| REEP1 | 0.007 | 1.176 | LAMP3 | 0.024 | 0.871 |
| GABRP | 0.009 | 0.909 | IGLV1_44 | 0.025 | 0.763 |
| SPIB | 0.01 | 0.715 | ATRNL1 | 0.028 | 1.155 |
| KRT17 | 0.011 | 0.863 | GABBR1 | 0.034 | 0.904 |
| RND1 | 0.015 | 1.284 | IGHA1 | 0.039 | 0.861 |
| GJB3 | 0.016 | 0.681 | SFRP1 | 0.044 | 0.919 |
| KRT23 | 0.016 | 0.892 |  |  |  |

Supplemented Table 4. Relative Importance of survival-related DEGs.

| **Gene** | **Importance** | **Relative Importance** | **Gene** | **Importance** | **Relative Importance** |
| --- | --- | --- | --- | --- | --- |
| PPP1R3C | 0.0042 | 1.0000 | GZMB | 0.0010 | 0.2406 |
| KRT23 | 0.0036 | 0.8587 | KRT17 | 0.0007 | 0.1784 |
| ALDH3B2 | 0.0033 | 0.7971 | IGLV1.44 | 0.0007 | 0.1772 |
| REEP1 | 0.0033 | 0.7899 | GABRP | 0.0004 | 0.0989 |
| SPIB | 0.0032 | 0.7556 | AR | 0.0000 | 0.0030 |
| CLGN | 0.0030 | 0.7120 | ATRNL1 | 0.0000 | -0.0045 |
| GLDC | 0.0024 | 0.5690 | LAMP3 | -0.0002 | -0.0400 |
| IGHM | 0.0022 | 0.5151 | IGHA1 | -0.0003 | -0.0692 |
| SFRP1 | 0.0015 | 0.3574 | GABBR1 | -0.0010 | -0.2312 |
| IGK | 0.0013 | 0.3147 | GJB3 | -0.0011 | -0.2704 |
| RND1 | 0.0011 | 0.2611 |  |  |  |

Supplemented Table 5. Multivariate analysis of candidate DEGs.

| **Gene** | **B** | **HR** | **95%CI** | ***P*** |
| --- | --- | --- | --- | --- |
| PPP1R3C | NS | | | |
| **KRT23** | -1.058 | 0.347 | 0.202-0.595 | <0.001 |
| **ALDH3B2** | 0.454 | 1.574 | 1.036-2.391 | 0.033 |
| **REEP1** | 0.513 | 1.670 | 1.111-2.510 | 0.014 |
| **SPIB** | -0.687 | 0.503 | 0.277-0.914 | 0.024 |
| CLGN | NS | | | |
| **GLDC** | -0.703 | 0.495 | 0.335-0.731 | <0.001 |
| IGHM | NS | | | |
